# Insights Into the Redox Sensitivity of *Chloroflexi* Hup-Hydrogenase Derived From Studies in *Escherichia coli*: Merits and Pitfalls of Heterologous [NiFe]-Hydrogenase Synthesis

**DOI:** 10.3389/fmicb.2018.02837

**Published:** 2018-11-21

**Authors:** Nadya Dragomirova, Patricia Rothe, Stefan Schwoch, Stefanie Hartwig, Constanze Pinske, R. Gary Sawers

**Affiliations:** Institute of Microbiology, Martin-Luther University Halle-Wittenberg, Halle, Germany

**Keywords:** hydrogen, formate, ferredoxin-like proteins, electron transfer, uptake hydrogenase, heterologous expression

## Abstract

The highly oxygen-sensitive hydrogen uptake (Hup) hydrogenase from *Dehalococcoides mccartyi* forms part of a protein-based respiratory chain coupling hydrogen oxidation with organohalide reduction on the outside of the cell. The HupXSL proteins were previously shown to be synthesized and enzymatically active in *Escherichia coli*. Here we examined the growth conditions that deliver active Hup enzyme that couples H_2_ oxidation to benzyl viologen (BV) reduction, and identified host factors important for this process. In a genetic background lacking the three main hydrogenases of *E. coli* we could show that additional deletion of genes necessary for selenocysteine biosynthesis resulted in inactive Hup enzyme, suggesting requirement of a formate dehydrogenase for Hup activity. Hup activity proved to be dependent on the presence of formate dehydrogenase (Fdh-H), which is typically associated with the H_2_-evolving formate hydrogenlyase (FHL) complex in the cytoplasm. Further analyses revealed that heterologous Hup activity could be recovered if the genes encoding the ferredoxin-like electron-transfer protein HupX, as well as the related HycB small subunit of Fdh-H were also deleted. These findings indicated that the catalytic HupL and electron-transferring HupS subunits were sufficient for enzyme activity with BV. The presence of the HupX or HycB proteins in the absence of Fdh-H therefore appears to cause inactivation of the HupSL enzyme. This is possibly because HupX or HycB aided transfer of electrons to the quinone pool or other oxidoreductase complexes, thus maintaining the HupSL heterodimer in a continuously oxidized state causing its inactivation. This proposal was supported by the observation that growth under either aerobic or anaerobic respiratory conditions did not yield an active HupSL. These studies thus provide a system to understand the redox sensitivity of this heterologously synthesized hydrogenase.

## Introduction

The bacterial genus *Dehalococcoides* belongs to the phylum *Chloroflexi* and the type species *D. mccartyi* is completely dependent on hydrogen for growth (Löffler et al., 2013; Schubert et al., 2018). *D. mccartyi* synthesizes several types of [NiFe]-hydrogenase (Hyd), and the hydrogen-uptake (Hup) hydrogenase is thought to be the main enzyme involved in H_2_-driven organohalide respiration. As *D. mccartyi* lacks quinones (Kube et al., 2005; Schipp et al., 2013), a direct transfer of the electrons derived from H_2_ oxidation by Hup via protein–protein interaction has been implicated (Kublik et al., 2016; Hartwig et al., 2017; Seidel et al., 2018). The Hup enzyme is found in a respiratory supercomplex comprising a two-subunit complex iron-sulfur molybdoprotein, OmeAB (organohalide molybdoenzyme) and one of a number of reductive dehalogenases (Rdh), which catalyze the reduction of particular organohalides that function as electron acceptors for the bacterium (Fincker and Spormann, 2017; Schubert et al., 2018). In addition, the ferredoxin-like protein HupX, which resembles electron-transferring subunits of oxidoreductases, is associated with the complex.

Hup comprises two structural components: the catalytic subunit HupL, containing the NiFe(CN)_2_CO cofactor and HupS, the small electron-transferring subunit, which is predicted to have three iron-sulfur clusters. The membrane-associated, ferredoxin-like protein HupX is encoded within the operon of the Hup hydrogenase, but seems to associate more tightly with the core OmeAB-Rdh complex (Hartwig et al., 2017; Seidel et al., 2018), suggesting that it is the main mediator of electron transfer and acts as a “connector” protein between HupSL and the rest of the complex. HupX is homologous to HybA, a component of the Hyd-2 H_2_-oxidizing hydrogenase of *Escherichia coli* (Sargent et al., 1998; Beaton et al., 2018) and recent studies have provided strong evidence indicating that HybA is responsible for coupling electron transfer to the quinone pool, as Hyd-2 has no membrane subunit with a recognized heme cofactor, necessary for electron transfer into the membrane (Dubini et al., 2002; Pinske et al., 2015; Beaton et al., 2018).

The ferredoxin-like family of electron–transfer proteins harbors four [4Fe-4S] clusters and an interaction network of several members of this family has been uncovered recently in *E. coli* (Pinske, 2018). One member is HycB, the small subunit of the formate dehydrogenase (Fdh-H) that forms one of the two catalytic centers of the formate hydrogenlyase (FHL) complex, and another is the related protein HydN, which is proposed to be involved in FHL complex assembly (Pinske, 2018). Generally, however, the physiological function of most members of this emerging superfamily of iron-sulfur-containing electron transfer proteins is not understood.

Due to the fact that *D. mccartyi* grows extremely slowly and produces limited amounts of biomass, making biochemical studies challenging, we have established a heterologous expression system for the synthesis of a functional Hup enzyme in *E. coli* (Hartwig et al., 2015b). It is hoped that this system will facilitate a detailed biochemical characterization of Hup. Despite the significant phylogenetic distance between *D. mccartyi* and *E. coli*, the Hyp maturation system responsible for [NiFe]-cofactor biosynthesis and insertion (Böck et al., 2006) is capable of recognizing the HupL apoprotein and generating an active enzyme when the complete operon encoding Hup is expressed under anaerobic conditions (Hartwig et al., 2015b).

**FIGURE 1:**
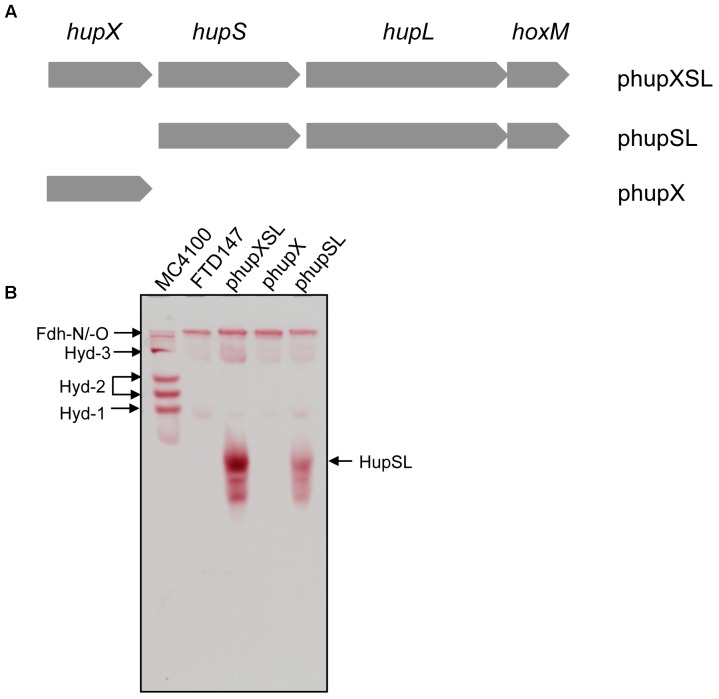
HupX is not required for heterologous HupSL activity. **(A)** Schematic representation of the plasmids used in this study is shown. The plasmid inserts are not drawn to scale but the complete *hupXSLhoxM* region encompasses 4216 bp (Hartwig et al., 2015b). **(B)** An in-gel activity stain for hydrogen-oxidizing activity is shown. Crude extracts (70 μg of protein; 30 μg in the case of wild type MC4100) derived from FTD147 (Δ*hyaB* Δ*hybC* Δ*hycE*) carrying the indicated plasmids were applied to a native polyacrylamide gel (7.5% w/v polyacrylamide). The migration positions of HupSL and the *E. coli* hydrogenases are indicated. The formate dehydrogenases Fdh-N and Fdh-O (Fdh-N/O) have a weak hydrogen-oxidizing activity (Soboh et al., 2011), which is also indicated and was used as an internal loading control for the experiment.

As well as the three structural genes, the *hupXSL-hoxM* operon (Figure 1A) also encodes a HupL-specific maturation endoprotease (HoxM). Initial characterization of the heterologously synthesized Hup enzyme identified a fast-migrating complex, mainly comprising HupS and HupL after native-PAGE, which migrated at a similar position as the complex present in crude extracts of *D. mccartyi* that contained HupSL and minor amounts of HupX (Hartwig et al., 2015b). This suggests that HupSL alone is capable of catalyzing H_2_-dependent reduction of the redox dye BV. The activity of the complex was oxygen-sensitive, even when synthesized anaerobically in the heterologous host (Hartwig et al., 2015b), suggesting that a cofactor in the enzyme is redox-sensitive. Whether this redox-sensitive cofactor is in HupL, HupS or HupX is unclear. Therefore, to address these questions, in the current study we decided to determine the conditions necessary for heterologous production of HupSL activity and whether any other components of the host’s metabolism, other than the Hyp proteins, are required for activity to be visualized. Surprisingly, we found a strong dependence for HupSL activity on the Fdh-H enzyme of the FHL complex. This dependence on Fdh-H for activity proved to be linked to an involvement of ferredoxin-like electron transfer proteins and to the redox sensitivity of the HupSL heterodimer.

## Materials and Methods

### Strains and Growth Conditions

The strains listed in Table 1 were used in this study. For routine molecular biology studies, growth was on LB-agar plates or in LB-broth at 37°C (Miller, 1972). Anaerobic growths were performed at 37°C as standing liquid cultures and cells were usually grown in M9 minimal medium (47.6 mM Na_2_HPO_4_ × 2 H_2_O, 22 mM KH_2_PO_4_, 8.4 mM NaCl, 20 mM NH_4_Cl, 2 mM MgSO_4_, 0.1 mM CaCl_2_, 0.1 mM thiamin dichloride, 0.2% w/v casamino acids) containing 0.8% (w/v) glucose, or 0.4% (v/v) glycerol plus 15 mM fumarate, or 0.8% (w/v) glucose plus 1% (w/v) nitrate, where indicated, as described (Sambrook et al., 1989). When growth in rich medium was performed, buffered TGYEP (1% w/v tryptone, 0.5% w/v yeast extract, 0.8% w/v glucose, 100 mM potassium phosphate, pH 6.5) was used (Begg et al., 1977). The growth medium was supplemented with trace element solution SLA (Hormann and Andreesen, 1989). When required, the antibiotic kanamycin or chloramphenicol was added to a final concentration of 50 or 25 μg ml^-1^, respectively. Cells were harvested anaerobically by centrifugation at 5,000 *g* for 15 min at 4°C when cultures had reached an OD_600__nm_ of between 0.8 and 1.2. Cell pellets were used immediately or stored at -20°C until use.

**Table 1:** Strains and plasmids used in this study.

Strain or plasmid	Relevant genotype or characteristic(s)	Reference or source
**STRAIN**		
MC4100	F^-^ *araD139* (*argF-lac*)*U169 ptsF25 deoC1 relA1 flbB5301 rspL150*	Casadaban, 1976
RM220	As MC4100, but Δ*pflAB*	Kaiser and Sawers, 1994
FTD147	As MC4100, but Δ*hyaB*Δ*hybC*Δ*hycE*	Redwood et al., 2008
FTD147Δ*fdnG*	As ^a^FTD147, but Δ*fdnG*	This work
FTD147Δ*fdoG*	As ^a^FTD147, but Δ*fdoG*	This work
FTD147Δ*fdhF*	As ^a^FTD147, but Δ*fdhF*	This work
FTD147Δ*fdnG*Δ*fdoG*	As ^a^FTD147, but Δ*fdnG* Δ*fdoG*	This work
FTD147Δ*fdnG*Δ*fdhF*	As ^a^FTD147, but Δ*fdnG* Δ*fdhF*	This work
FTD147Δ*fdoG*Δ*fdhF*	As ^a^FTD147, but Δ*fdoG* Δ*fdhF*	This work
FTD147Δ*fdnG*Δ*fdoG*Δ*fdhF*	As ^a^FTD147, but Δ*fdnG* Δ*fdoG* Δ*fdhF*	This work
FTD147 Δ*selC*	As FTD147, but Δ*selC* Kan^R^	This work
FTD150	As MC4100, but Δ*hyaB*Δ*hybC*Δ*hycE* Δ*hyfG*	Redwood et al., 2008
FTD150Δ*selB*	As FTD150, but Δ*selB* Kan^R^	This work
CP1170	As MC4100, but Δ*hyaB*Δ*hybC*Δ*hycA-I*	This work
CPH008	As MC4100, but Δ*hycA-I ΔfdhF*	This work
CPH020	As MC4100, but Δ*hyaB*Δ*hybC*Δ*hycA-I*Δ*fdhF*	Pinske, 2018
CPH021	As MC4100, but Δ*hyaB*Δ*hybC*Δ*hycA-I*Δ*fdhE*	This work
RT2	As MC4100, but Δ*hyaB*Δ*hybC*Δ*hycA-I*Δ*fdhE*Δ*pflA*	Pinske and Sargent, 2016
**PLASMIDS**		
pCP20	*FLP*^+^, λ*cI857*^+^, λ*p_R_* Rep^ts^, Amp^R^, Cm^R^	Cherepanov and Wackernagel, 1995
^b^pSHH18	pACYC-Duet-I, *hupXSLhoxM*^+^ Cm^R^ = phupXSL	Hartwig et al., 2015b
phupSL	pACYC-Duet-I, *hupSLhoxM^+^*, Cm^R^	This work
phupX	pACYC-Duet-I, *hupX*^+^, Cm^R^	This work

^a^*The series of FTD147 mutants with different combinations of Fdh gene mutations was constructed by transduction of mutations from the corresponding Keio collection of mutants (see Materials and Methods). ^*b*^Note that for reasons of clarity, this plasmid was referred to as phupXSL throughout this study.*

### Plasmid and Strain Construction

The *hupX* gene (*cbdbA131*) was amplified as a 1212 bp DNA fragment from chromosomal DNA isolated from *D. mccartyi* strain CBDB1 using Pfu DNA polymerase and the oligonucleotides hupX_fw (5′-GGGGCATATGCCTAATGGAATGCTGATTG-3′) and hupX_re (5′-GGGGCTCGAGCTAGTGCTTGCCAGCCTTG-3′) and cloned in plasmid pACYC-Duet-I. Plasmid phupSL was constructed by using pSHH18 (referred to as phupXSL throughout this study) as template in a PCR mutagenesis employing the Q5^®^ Site-Directed Mutagenesis Kit (New England Biolabs, NEB). Care was taken when deleting the *hupX* gene to ensure that the ribosome binding site for the downstream *hupS* gene remained intact by using the oligonucleotides hupSLhoxM_fw (5′-ATGGAGTAGGλAATGTTTAATAC-3′) and hupSLhoxM_re (5′-TCCTGTTGCCCCCCTTGT-3′) and by following the instructions given in the Q5^®^ Site-Directed Mutagenesis Kit.

*E. coli* strains were constructed using P1*kc*-mediated phage transduction (Miller, 1972) to introduce the respective defined deletion mutation from the appropriate donor strain obtained from the Keio collection (Baba et al., 2006) to generate the series of FTD147 mutants lacking the structural genes encoding the three formate dehydrogenases of *E. coli*. When multiple gene knock-outs were constructed, the plasmid pCP20 was used to remove the kanamycin antibiotic resistance cassette as described (Cherepanov and Wackernagel, 1995).

### Preparation of Crude Cell Extracts and Cell Fractionation

Unless otherwise stated, all experiments were performed in an anaerobic Coy^TM^ chamber under an atmosphere of 95% nitrogen/5% hydrogen. For standard hydrogenase enzyme activity determination, *E. coli* cell paste was re-suspended at a ratio of 1 g cell wet weight to 3 ml 50 mM MOPS buffer, pH 7. Cells were disrupted by sonication (30 W power for 5 min with 0.5 s pulses). Unbroken cells and cell debris were removed by centrifugation for 30 min at 50,000 *g* and 4°C. The resulting crude extract, unless otherwise stated, was used for all studies reported herein.

In order to perform sub-cellular fractionation, periplasmic, soluble and membrane fractions were isolated as described (Sawers et al., 1985).

Determination of protein concentration was done as described (Lowry et al., 1951).

### Non-denaturing Polyacrylamide Gel Electrophoresis and Activity-Staining

Unless otherwise specified, non-denaturing polyacrylamide gel electrophoresis (PAGE) was performed anaerobically. Separating gels included 0.1% (w/v) Triton X-100 as described (Ballantine and Boxer, 1985). The crude extracts, or sub-cellular fractions, were incubated with a final concentration of 4% (w/v) Triton X-100 prior to application (usually 50 μg of protein) to the gel, which included 6% (w/v) polyacrylamide. Hydrogenase activity-staining was done in 50 mM MOPS buffer pH 7.0, as described (Sawers et al., 1985; Pinske et al., 2012), and included 0.5 mM BV and 1 mM 2,3,5-triphenyltetrazolium chloride (TTC). Gels were incubated under an atmosphere of 100% hydrogen gas.

### Hydrogenase Activity Assay

Measurement of hydrogenase enzyme activity using BV as electron acceptor was performed as described (Ballantine and Boxer, 1985; Pinske et al., 2011). Briefly, anaerobically prepared cuvettes (1.6 ml) were filled with 0.8 ml of H_2_-saturated, anaerobic 50 mM MOPS buffer, pH 7.0, including 4 mM BV and placed under a H_2_ atmosphere. After baseline determination, the assay was initiated by adding enzyme sample (approximately 150 μg of protein). All assays were performed at 25°C. The wavelength used was 600 nm and an ε_M_ value of 7400 M^-1^ cm^-1^ was assumed for reduced BV. One million unit of enzyme activity corresponded to the reduction of 1 nmol of substrate min^-1^. Enzyme assays were performed in triplicate using three biological replicates.

### Denaturing Polyacrylamide Gel Electrophoresis (PAGE) and Western Blotting

Polypeptides in crude extracts were separated by 12.5% (w/v) sodium dodecyl sulfate (SDS)-PAGE (Laemmli, 1970) and gels were either stained with Coomassie Brilliant Blue R or transferred to nitrocellulose membranes for western blotting, which was performed as described (Towbin et al., 1979). The antibodies used were either anti-Strep-tag (IBA Life Sciences), anti-Hyd-2 (Sargent et al., 1998), anti-HupL or anti-HupX peptide antibodies (Hartwig et al., 2017).

## Results

### HupL and HupS Are Sufficient for BV Reduction Activity

The *hupXSLhoxM* operon has been shown to be functional in anaerobically grown *E. coli* (Hartwig et al., 2015b). In order to determine whether all three structural components (HupSL and HupX) are essential for the manifestation of the H_2_:BV oxidoreductase activity observed in that study, we constructed two additional plasmid derivatives, one of which carried only the *hupX* gene, while the other included *hupSLhoxM* but lacked *hupX* (Figure 1A). These plasmids, along with pSHH18 (*hupXSLhoxM*^+^; Hartwig et al., 2015b; here referred to as phupXSL in the aid of clarity), were introduced into FTD147, which lacks the genes encoding the catalytic subunits of Hyd-1, Hyd-2, and Hyd-3 (Redwood et al., 2008). After fermentative growth, crude extracts were separated in native-PAGE and stained for hydrogenase enzyme activity (Figure 1B). As anticipated, the plasmid encoding only HupX showed no hydrogenase enzyme activity in extracts of strain FTD147 (Δ*hyaB*Δ*hybC*Δ*hycE*), while both of the other plasmids resulted in a fast-migrating activity band corresponding to the HupSL heterodimer (Figure 1B). Notably, although the activity resulting from introduction of the plasmid lacking the *hupX* gene (phupSL in Figure 1B) was apparently weaker than that resulting from introduction of phupXSL, both enzyme activities showed very similar migration characteristics, indicating that HupX is neither necessary for the ability of the enzyme to reduce BV nor seems to co-migrate with HupSL in this particular activity band. This result correlates well with earlier mass spectrometric analyses of heterologously expressed enzyme, which identified mainly the HupL protein (Hartwig et al., 2015b).

### Manifestation of Heterologous HupSL Enzyme Activity Requires Fermentative Growth Conditions

**FIGURE 2:**
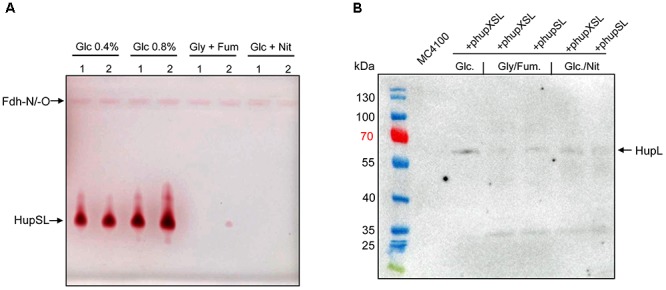
Heterologous HupSL enzyme activity is only detectable in fermentatively growing *E. coli* cells. **(A)** Crude extracts derived from FTD147 (Δ*hyaB*Δ*hybC*Δ*hycE*) transformed with plasmid phupXSL (1) or phupSL (2) after growth in M9 minimal medium with the indicated carbon sources were separated in native-PAGE and stained for hydrogenase enzyme activity, as described in the legend to Figure 1. **(B)** A western blot using peptide antibodies raised against HupL (Hartwig et al., 2017) is shown in which the same extracts used in part A were separated in 10% (w/v) SDS-PAGE. The molecular mass marker proteins are presented in kDa. MC4100 was the wild type strain.

In order to optimize conditions for the analysis of heterologously produced HupSL activity, we tested different anaerobic growth conditions using FTD147 (Δ*hyaB*Δ*hybC*Δ*hycE*) transformed with either phupXSL or phupSL (Figure 2A). The activity band was slightly more intense when cells were grown with 0.8% w/v glucose compared with half that glucose concentration (0.4% w/v). Suprisingly, however, no HupSL activity could be detected when cells were grown under anaerobic respiratory conditions, with either glycerol and fumarate or glucose and nitrate. Western blot analysis of the extracts derived from anaerobically grown strains after separation by SDS-PAGE using peptide antibodies raised against HupL revealed that the HupL polypeptide could be detected in each extract (Figure 2B). This indicates that a lack of transcription of the *hup* genes under respiratory conditions was not the reason for absence of HupSL enzyme activity. Surprisingly, it was not possible to restore *in vitro* HupSL enzyme activity to these extracts, even by incubating the extracts under reducing conditions. This suggests that the HupSL enzyme was irreversibly inhibited under the oxidizing conditions that prevailed within the cells grown under these conditions.

Quantitative assessment of H_2_-dependent BV reduction activity in anaerobically prepared, concentrated crude extracts of FTD147 (Δ*hyaB*, Δ*hybC*, Δ*hycE*) transformed with phupXSL measured a low but detectable hydrogenase activity of approximately 60 mU/mg (Table 2), which is in good agreement with previously determined HupSL activity in *E. coli* extracts (Hartwig et al., 2015b). The phenotypically identical strain CP1170 (Δ*hyaB*, Δ*hybC*, Δ*hycE*) had a background activity of 10 mU/mg. Brief incubation of the extract from FTD147 + phupXSL in the presence of oxygen resulted in a reduction of the HupSL activity by 50% (Table 2).

**Table 2:** H_2_:BV oxidoreductase activities of *E. coli* strains carrying phupXSL.

Strain	Anaerobic hydrogenase activity (mU mg protein^-1^)	Activity after O_2_ exposure (mU mg protein^-1^)^a^
CP1170 (Δ*hyaB*,Δ*hybC*,Δ*hycA-I*)	10 ± 3^b^	n.d^c^
FTD147 (Δ*hyaB*,Δ*hybC*,Δ*hycE*) + phupXSL	58 ± 18	29 ± 9
FTD147Δ*fdnG*,Δ*fdoG*	26 ± 13	n.d.
FTD147Δ*fdnG*,Δ*fdoG* + phupXSL	59 ± 1	22 ± 1
FTD147Δ*fdhF*	4 ± 2	n.d.
FTD147Δ*fdhF* + phupXSL	11 ± 1	n.d

^a^*Crude extracts were exposed to air for 15 min prior to determination of hydrogenase activity. ^*b*^Assays were performed in triplicate from 3 independent growth experiments. The activity is shown together with the standard deviation. ^*c*^n.d, not determined.*

### HupSL Activity in *E. coli* Requires a Functional Selenocysteine-Insertion Machinery

**FIGURE 3:**
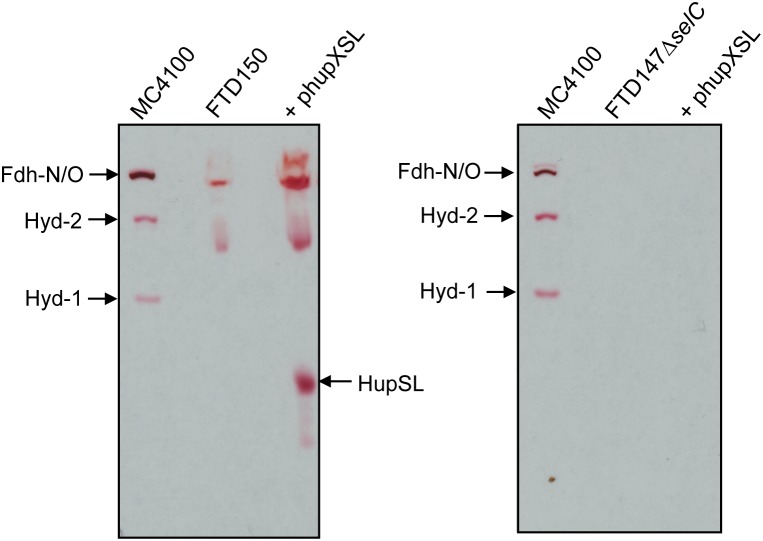
Appearance of HupSL enzyme activity is dependent on a functional selenocysteine biosynthetic apparatus. Crude extracts (100 μg of protein, except M4100 where 30 μg of protein were applied) of the indicated strains were separated in native-PAGE and stained for hydrogenase enzyme activity as described in the legend to Figure 1. Strains used include: MC4100 (wild type); FTD150 (Δ*hyaB*Δ*hybC*Δ*hycE* Δ*hyfG*); FTD147Δ*selC* (Δ*hyaB*Δ*hybC*Δ*hycE* Δ*selC*).

The lack of HupSL enzyme activity after respiratory growth is reminiscent of the effects of these growth conditions on appearance of *E. coli* Hyd-3 and Fdh-H enzyme activities (Sawers et al., 1985), with the exception that the effect on synthesis of the *E. coli* enzymes is at the transcriptional level due to depletion of the regulatory metabolite formate (Rossmann et al., 1991). Due to the fact that HupSL is naturally associated with a formate dehydrogenase-like enzyme, OmeAB (Kublik et al., 2016; Hartwig et al., 2017), we wished to examine the influence of the Fdh-O and Fdh-N enzymes, which are phylogenetically related to OmeAB, on HupSL enzyme activity. Initially, we introduced into strain FTD147 a mutation in the *selC* gene, which encodes the selenocysteinyl-tRNA_Sec_ necessary for translation of special UGA codons as selenocyteine, and which, when deleted, renders all Fdhs inactive (Leinfelder et al., 1988). This would provide information on whether HupSL enzyme activity was influenced by defects in formate metabolism. The left panel shown in Figure 3 shows a control for HupSL activity revealing that is was readily detectable in strain FTD150 (Δ*hyaB*Δ*hybC*Δ*hycE*Δ*hyfG*), which is identical to strain FTD147 with the exception that the gene encoding the catalytic subunit of Hyd-4 (Andrews et al., 1997) is also deleted (Redwood et al., 2008). Thus, both FTD150 and FTD147 yield an identical phenotype with regard to the heterologous HupSL activity (see also below). Analysis of an extract of the FTD147 Δ*selC* mutant revealed that no HupSL activity was detectable (Figure 3 right panel). The lack of *selC* was confirmed by the absence of the H_2_:BV oxidoreductase activity associated with Fdh-N/O in the strain (Soboh et al., 2011). This result confirms that HupSL enzyme activity is linked to formate metabolism, most likely through one of the formate dehydrogenases (Fdh) the bacterium synthesizes under anaerobic conditions. Introduction of a mutation in *selB*, which encodes the special translation factor required to decode the UGA codon as selenocysteine (Forchhammer et al., 1989), into FTD150 also revealed a similar lack of HupSL activity (data not shown), confirming that the phenotype was due to a lack of selenocysteine incorporation.

### In-Gel HupSL Activity Depends on the Fdh-H Enzyme

**FIGURE 4:**
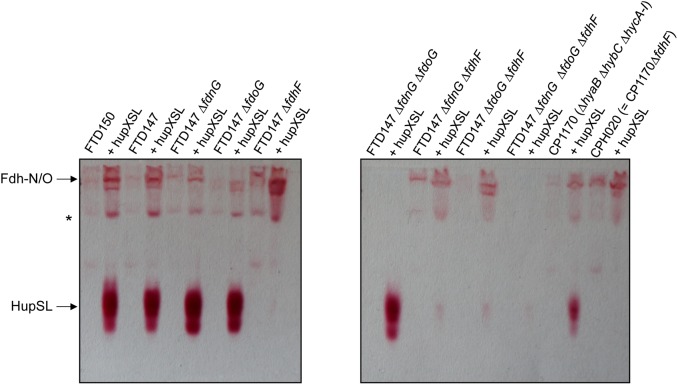
Only strains lacking Fdh-H, the product of the *fdhF* gene, failed to show HupSL enzyme activity. Crude extracts (90 μg of protein) of the indicated strains were separated in native-PAGE and stained for hydrogenase enzyme activity as described in the legend to Figure 1. The asterisk denotes a fast-migrating form of the Fdh-O enzyme (Hartwig et al., 2015a). Strains used include: FTD147 (Δ*hyaB*Δ*hybC*Δ*hycE*), plus its deletion derivatives; FTD150 (Δ*hyaB*Δ*hybC*Δ*hycE* Δ*hyfG*); CP1170 (Δ*hyaB*Δ*hybC*Δ*hycA-I*); CPH020 (Δ*hyaB*Δ*hybC*Δ*hycA-I*Δ*fdhF*).

Dependence on the selenocysteine biosynthetic machinery for appearance of HupSL enzyme activity suggests an involvement of one or more of the three Fdhs present in *E. coli*. To determine which of the three Fdhs is required for the appearance of heterologous HupSL activity, we constructed a series of strains (see Table 1) lacking one or more of the genes encoding the catalytic subunit of FdnG (of Fdh-N), FdoG (of Fdh-O), or FdhF (of Fdh-H) (Pinske and Sawers, 2016; Figure 4). Strain FTD150, which lacked all four hydrogenases and the quadruple and quintuple mutants of FTD147, which lacked Hyd-1, Hyd-2, Hyd-3 as well as either or both respiratory Fdhs (Fdh-N and Fdh-O), retained fully active HupSL (Figure 4). Hydrogenase activity in extracts derived from FTD147Δ*fdnG*Δ*fdoG* with plasmid phupXSL was approximately 60 mU, while the strain without plasmid had approximately half this activity (Table 2). Exposure of the crude extract from FTD147Δ*fdnG*Δ*fdoG* transformed with phupXSL to air resulted in a similar 50–60% reduction in hydrogenase activity as was observed with FTD147 containing phupXSL (Table 2). This result confirms that HupSL is oxygen-labile (Hartwig et al., 2015b).

The only strains that lacked a detectable HupSL enzyme activity band were those that lacked the *fdhF* gene, which encodes the Fdh-H component of the FHL complex (Figure 4). Assay of hydrogenase activity in extracts derived from FTD147Δ*fdhF* + phupXSL failed to show HupSL-dependent hydrogenase activity (Table 2). These findings indicate that for full HupSL activity to be manifested, an active Fdh-H enzyme is required.

**FIGURE 5:**
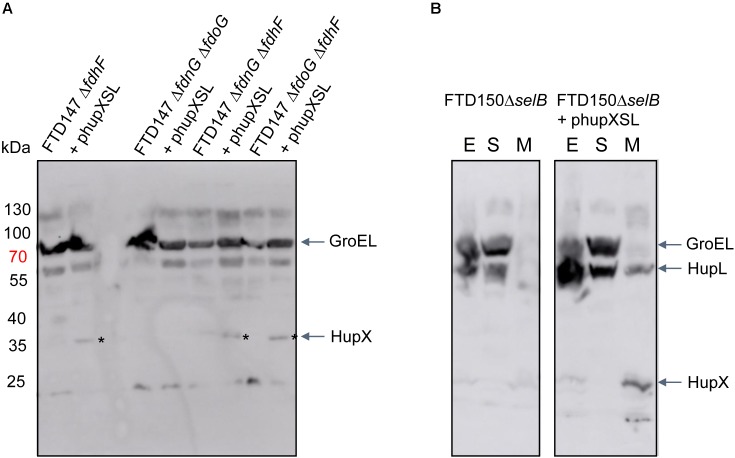
HupX is only detectable in extracts of strains lacking the *fdhF* gene. Western blots using peptide antibodies raised against HupX or HupL are shown in which 50 μg of crude extracts **(A)** or subcellular fractions **(B)** were separated in 10% (w/v) SDS-PAGE. The migration positions of molecular mass marker proteins are shown in kDa. The strong signal migrating around 70 kDa represents GroEL, which cross-reacted with the HupX antibodies, and acted as a protein loading control. The asterisks denote the HupX polypeptide. In **(B)**, the letters E, S, and M represent crude extract, soluble cytoplasmic fraction and membrane fraction, respectively. Strain used include: deletion derivatives of FTD147 (Δ*hyaB*Δ*hybC*Δ*hycE*); and deletion derivatives of FTD150 (Δ*hyaB*Δ*hybC*Δ*hycE* Δ*hyfG*).

In order to determine what the link between the appearance of HupSL enzyme activity and Fdh-H might be, we first performed a western blot using anti-HupX antibodies and with crude extracts derived from some of the strains shown in Figure 4. Surprisingly, HupX was only detectable in extracts of strains lacking *fdhF*, which encodes the Fdh-H enzyme, and, as expected, only in those strains carrying the phupXSL plasmid (Figure 5). This suggests that when Fdh-H was absent, HupX was stably synthesized and when Fdh-H was present in the cells, HupX became unstable and was presumably degraded.

### The Influence of Fdh-H on HupSL Activity Is Indirect via the Ferredoxin-Like Proteins HupX and HycB

**FIGURE 6:**
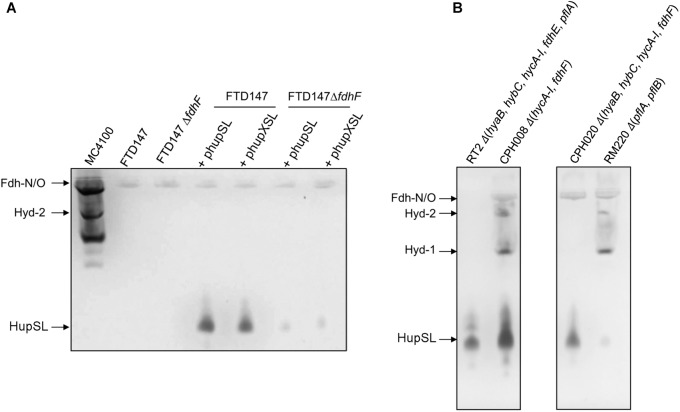
Deletion of the *hycA-I* operon restores HupSL activity in a *fdhF* mutant. **(A)** Crude extracts derived (approximately 60 μg of protein) from the indicated strains after growth in M9 minimal medium with 0.8% (w/v) glucose were separated in native-PAGE and stained for hydrogenase enzyme activity, as described in the legend to Figure 1. In **(B)**, strains were transformed with plasmid phupSL. The migration positions of the respective hydrogen-oxidizing enzymes are indicated. Strain used include: MC4100 represents wild type; FTD147 (Δ*hyaB*Δ*hybC*Δ*hycE*); FTD147 Δ*fdhF*; RT2 (Δ*hyaB*Δ*hybC*Δ*hycA-I*Δ*fdhE*Δ*pflA*); CPH008 (Δ*hycA-I ΔfdhF*); CPH020 (Δ*hyaB*Δ*hybC*Δ*hycA-I*Δ*fdhF*); RM220 (Δ*pflA*Δ*pflB*).

To examine whether HupX might influence HupSL activity, plasmids phupSL and phupXSL, both encoding HupL, HupS and the endoprotease HoxM, but only the latter also encoding HupX (Figure 1A), were introduced into strains FTD147 and FTD147Δ*fdhF* and enzyme activity was compared after anaerobic growth with glucose (Figure 6A). The products of both plasmids in strain FTD147Δ*fdhF* showed a strongly reduced activity of HupSL compared with the *fdhF*^+^ strain FTD147. This result indicates that the dependence on Fdh-H for HupSL activity was retained in the absence of HupX.

A recent study in *E. coli* identified a flexible interaction network of ferredoxin-like proteins with Fdh-H, including its small, electron-transferring subunit, HycB (Pinske, 2018). HycB and HupX belong to this family but share only 24% amino acid identity (38% similarity) in a MuscleWS alignment. The HupX protein, however, cannot functionally replace HycB in formate-dependent BV reduction (data not shown). Due to the link between HupSL activity and Fdh-H demonstrated above, we therefore examined whether the presence of HycB influenced HupSL’s ability to reduce BV. To do this, we analyzed HupSL activity in strain CP1170 (Δ*hyaB*, Δ*hybC*, Δ*hycA-I*), which is similar to FTD147 (Δ*hyaB*, Δ*hybC*, Δ*hycE*) with the exception that the complete *hyc* operon is deleted in CP1170, rather than only the *hycE* gene (Table 1). Introduction of plasmid phupXSL into CP1170 and its Δ*fdhF* derivative CPH020 (Δ*hyaB*Δ*hybC*Δ*hycA-I*Δ*fdhF*) (Table 1), revealed that the dependence on Fdh-H for HupSL activity was retained (Figure 4, right side of right panel). However, introduction of plasmid phupSL (lacking *hupX*) into CPH020 (CP1170 Δ*fdhF*) revealed that HupSL activity in native PAGE was no longer reduced in the absence of Fdh-H (Figure 6B, lane 1, right panel). Introduction of phupSL into strain CPH008 (Δ*hycA-I, ΔfdhF*) in which Hyd-1 and Hyd-2 are still active, but all structural components of the FHL complex are missing, demonstrated that HupSL activity was retained, and even slightly more intense (Figure 6B, lane 2 left panel). As a final control, we analyzed HupSL activity after introduction of phupSL into strain RT2 (Δ*hyaB*Δ*hybC*Δ*hycA-I*Δ*fdhE*Δ*pflA*), which lacks Hyd-1, Hyd-2 and Hyd-3, as well as Fdh-N/O (through the *fdhE* mutation; Mandrand-Berthelot et al., 1988; Lüke et al., 2008) and the formate-inducible Fdh-H, due to the lack of active PflB (due to the *pflA* mutation), which is required for formate production (Sawers and Böck, 1988; Rossmann et al., 1991). This strain was chosen because it uses a different combination of mutations to generate the same phenotype, i.e., no hydrogenase or formate dehydrogenase enzymes, and no HycB protein. HupSL activity was also retained in this genetic background confirming that strains devoid of HupX and HycB exhibit HupSL- and H_2_-dependent reduction of BV (Figure 6B, lane 1).

Surprisingly, however, when phupSL was introduced into strain RM220 (Δ*pflAB*), which generates considerably reduced levels of formate under respiratory conditions (Suppmann and Sawers, 1994), and thus expresses only low levels of the formate-inducible *hyc* operon (Rossmann et al., 1991), the anticipated high activity of HupSL was not observed (Figure 6B, lane 4). This result indicates that even the low levels of HycB produced in this strain (Rossmann et al., 1991) are likely sufficient to inhibit HupSL activity.

## Discussion

In this study, we analyzed how the fermentative metabolism of the *E. coli* host influenced the redox dye-reducing activity of a heterologously synthesized hydrogen-uptake [NiFe]-hydrogenase from the *Chloroflexi* phylum. We had previously demonstrated that the host’s Hyp-maturation machinery was capable of recognizing the large subunit precursor pre-HupL and of successfully introducing the bimetallic [NiFe]-cofactor, generating active enzyme (Hartwig et al., 2015b). We also showed in that particular study that the HupSL hydrogenase had H_2_:BV oxidoreductase activity, which could be identified after anaerobic native-PAGE. Here we made the surprising discovery that the appearance of this HupSL enzyme activity was apparently dependent on whether the host’s Fdh-H enzyme was synthesized or not. Under conditions favoring synthesis of Fdh-H, HupSL activity was observed, while in strains unable to synthesize Fdh-H, due to a deletion of the *fdhF* structural gene or the selenocysteine insertion machinery, no, or substantially reduced, activity was detected. Notably, however, this lack of enzyme activity did not result from a lack of synthesis of the HupSL enzyme, but rather appears to be due to an inactivation of the enzyme.

**FIGURE 7:**
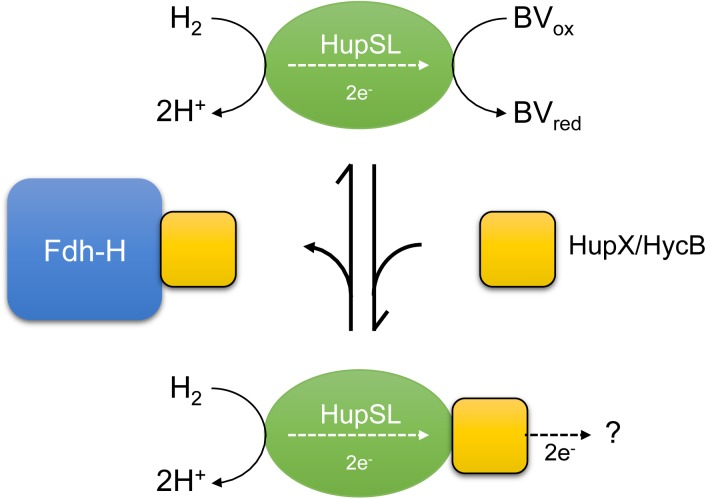
A schematic depiction of the current interpretation of our data is shown. The hypothesis states that the HupSL heterodimer is capable of interacting with and reducing BV and if HupX or HycB are free and not sequestered by Fdh-H they prevent HupSL transferring electrons to BV and instead deliver them to currently unknown acceptor complexes, or possibly to the quinone pool (?). HupX has been shown to interact with HupSL (Hartwig et al., 2017; Seidel et al., 2018), while this has not been so far shown for HycB.

A recent study revealed that Fdh-H interacts with at least three electron-transferring small subunits, all of which belong to the ferredoxin-like family of electron-transfer proteins and possibly facilitate the coupling of Fdh-H with different enzyme complexes (Pinske, 2018). We currently interpret our data to indicate that the apparent dependence on Fdh-H for HupSL activity is, in fact, indirect and likely due to Fdh-H sequestering these small subunits, in particular HycB of the FHL complex. Notably, the HupX protein, which is presumed to mediate electron transfer within the Hup-Ome-Rdh supercomplex in the natural host *D. mccartyi* (Schubert et al., 2018), also belongs to the ferredoxin-like superfamily and this protein’s ability to interact with the HupSL heterodimer also appears to be influenced by the presence of Fdh-H. If Fdh-H is either genotypically or phenotypically (e.g., through strongly reduced formate synthesis; Rossmann et al., 1991) absent, the ferredoxin-like proteins HupX or HycB remain consequently unbound within the cell. We suggest that if HupX is also absent, HycB can transiently interact with or modulate the HupSL enzyme within the cell prior to separation in the native-PAGE, rendering the enzyme inactive. This inactivity could result from a loss of the ability of the heterodimer to reduce or interact with BV in the presence of H_2_. Alternatively, these ferredoxin-like proteins might act by sequestering the HupSL complex resulting in an inactive complex in the native-PAGE; or indeed a combination of both effects might be the cause (Figure 7). The consequence would be that HupSL activity becomes visible in the absence of HycB despite simultaneous absence of Fdh-H. The redox-potentials of the ferredoxin-like proteins have not yet been determined.

The model shown in Figure 7 presents a working hypothesis for how we currently interpret our data. Under fermentative growth conditions Fdh-H is present and is available to interact with HycB. The presence of Fdh-H also prevents HupX interacting with HupSL, possibly forming an interaction with HupX, allowing the HupSL heterodimer to interact with and reduce BV.

How does an ability to interact with HupX, and possibly HycB, interfere with electron transfer to BV by HupSL? The redox dye BV can accept electrons directly from the electron-transferring subunit HupS, as evidenced by the fact that deletion of the *hupX* gene does not significantly affect H_2_-dependent reduction of BV by HupSL. Moreover, previous mass spectrometric analysis of the Hup activity band isolated after native-PAGE revealed mainly HupL and HupS to be present, suggesting that HupX’s interaction with the heterodimer is weak or transient (Hartwig et al., 2015b). Moreover, in the natural host *D. mccartyi*, HupX preferentially associates with OmeAB, the formate dehydrogenase homolog, and reductive dehydrogenases rather than with HupSL (Hartwig et al., 2015b; Seidel et al., 2018; Dragomirova and Sawers, unpublished observations), which supports the suggestion that the affinity of HupX for HupSL is low. We also observed using antibodies raised against HupX that it is only readily detectable in the membrane fraction of cells that lack Fdh-H, suggesting that when Fdh-H is present it is more readily degraded. How this apparent degradation is controlled is currently unclear.

The HupS subunit encodes a functional Tat-signal peptide allowing its transport across the cytoplasmic membrane (Hartwig et al., 2015b). Together with HupX, the HupSL complex could be sufficiently anchored in the membrane to transfer the electrons derived from oxidation of H_2_ to the quinone pool, which is also the function of another HupX homolog, the HybA protein of *E. coli* Hyd-2 (Pinske et al., 2015; Beaton et al., 2018).

Support for the oxidative inactivation of HupSL was provided by the demonstration of inactivation of the enzyme complex after growth of the *E. coli* host under respiratory conditions, with either O_2_ (*E*^o^′ = +830 mV), NO_3_^-^ (*E*^o^′ = +420 mV), or fumarate (*E*^o^′ = 0 mV) as electron acceptor. We have only been able to detect HupSL activity after fermentative growth (*E*^o^′ = -415 mV), strongly suggesting that the enzyme retains activity only under strongly reducing conditions, which are also likely to be those prevailing in the environmental conditions where *D. mccartyi* is found (Löffler et al., 2013).

These studies thus provide a platform to study how heterologously synthesized hydrogenases can be integrated into the host’s anaerobic metabolism. Clearly, this work is at an early stage but one of the next steps will be to examine whether electrons derived from H_2_ oxidation can be coupled to reduction of *E. coli*-typical electron acceptors, e.g., fumarate. Initial studies examining hydrogen-driven fumarate reduction by HupXLS yielded first indications that a weak, but unfortunately so far irreproducible, activity was detectable (Schwoch et al., unpublished data). Optimization of Hup enzyme synthesis and membrane integration will likely be required for this approach to be fruitful.

Because *D. mccartyi* species are not amenable to large-scale biochemical analysis, and are currently genetically intractable, using the *E. coli* Hyd- and Fdh-negative host strains developed here will provide a means of studying the biochemical mechanism(s) underlying the loss of HupSL activity in response to oxidizing redox conditions and whether this effect is linked to a particular iron-sulfur cluster, or clusters, in HupS, or whether the bimetallic cofactor in HupL is the target of irreversible inactivation. A recent study by Hartmann et al. (2018) indicates that, at least for certain [NiFe]-hydrogenases, the NiFe(CN)_2_CO cofactor is not sensitive to oxidative conditions, suggesting that it might indeed be the electron-transfer pathway that is disrupted by non-reducing redox potentials.

## Author Contributions

ND, PR, SS, SH, and CP designed and performed the experiments and analyzed the data. CP and RS conceived the study, interpreted the data and drafted the manuscript. All authors read and approved the manuscript.

## Conflict of Interest Statement

The authors declare that the research was conducted in the absence of any commercial or financial relationships that could be construed as a potential conflict of interest.
